# Interaction of Tau construct K18 with model lipid membranes†

**DOI:** 10.1039/d1na00055a

**Published:** 2021-06-17

**Authors:** Mehdi Azouz, Cécile Feuillie, Michel Lafleur, Michaël Molinari, Sophie Lecomte

**Affiliations:** Institute of Chemistry and Biology of Membranes and Nano-Objects, CNRS, Université de Bordeaux, INP Bordeaux, UMR5248 allée Geoffroy Saint Hilaire 33600 Pessac France cecile.feuillie@u-bordeaux.fr sophie.lecomte@u-bordeaux.fr; Department of Chemistry, Université de Montréal Succursale Centre-Ville Montréal C.P. 6128 Québec Canada H3C 3J7

## Abstract

One of the hallmarks of Alzheimer's disease (AD) is the formation of neurofibrillary tangles, resulting from the aggregation of the tubulin associated unit protein (Tau), which holds a vital role in maintaining neuron integrity in a healthy brain. The development of such aggregates and their deposition in the brain seem to correlate with the onset of neurodegeneration processes. The misfolding and subsequent aggregation of the protein into paired helical filaments that further form the tangles, lead to dysfunction of the protein with neuronal loss and cognitive decline. The aggregation of the protein then seems to be a causative factor of the neurodegeneration associated with AD. The hypothesis of an involvement of the membrane in modulating the misfolding and assembly of Tau into paired helical filaments attracts increasing interests. To provide more insight about how lipids can modulate the interactions with Tau, we have conducted a comprehensive Atomic Force Microscopy (AFM) study involving supported lipid bilayers of controlled compositions with the Tau microtubule-binding construct K18. Particularly, the effects of zwitterionic and negatively charged phospholipids on the interaction have been investigated. Deleterious solubilization effects have been evidenced on fluid zwitterionic membranes as well as an inability of K18 to fragment gel phases. The role of negative lipids in the aggregation of the peptide and the particular ability of phosphatidylinositol-4,5-bisphosphate (PIP_2_) in inducing K18 fibrillization on membranes are also reported.

## Abstract 2

AFM imaging of model membranes of controlled composition in interaction with Tau construct K18 showed PIP_2_ induced K18 fibrillization.

## Graphical abstract

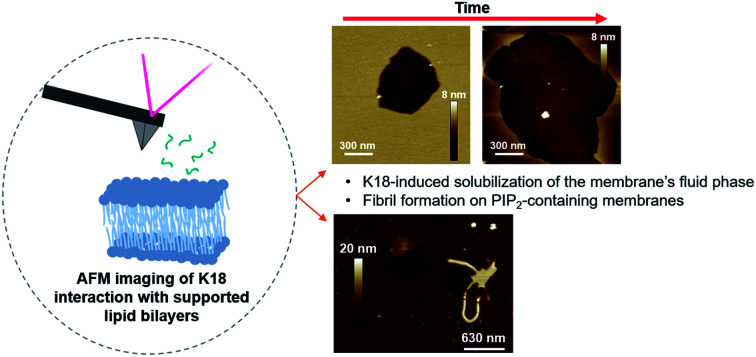

## Introduction

The tubulin associated unit, classically abbreviated Tau, is a protein that binds to microtubules and ensures their stability, thus participating in maintaining the cytoskeleton integrity and the viability of neuron cells.^1,2^ Pathological modifications of the protein's native state are thought to impair the tau/microtubule binding, and are involved in a wide range of neurodegenerative disorders called tauopathies, including Alzheimer's disease (AD).^3,4^ One of the hallmarks of AD is the presence of extracellular aggregates of Aβ amyloid, as well as intracellular aggregates of Tau called neurofibrillary tangles (NFT).^3^ Prior to forming highly structured NFT, Tau self-assembles into intertwined strands and fibrils called paired helical filaments (PHF), through which the protein expresses amyloid-like structures.^5–7^ The aggregation of Tau into PHF is suspected to prevent the protein's association to microtubules, eventually leading to the collapsing and death of neurons. The hyperphosphorylated state of Tau within PHF suggests that a molecular modification of the protein is associated with its aggregation.^8,9^ Yet, it remains unclear whether the aggregation pattern stems from the phosphorylation process.

Tau aggregation and accumulation in the brain are more temporally correlated to changes in cognition characteristics of AD, contrarily to Aβ.^10,11^ Hence, getting insights into the mechanistic process of Tau aggregation *in vitro* is of paramount importance to better understand mechanisms that might induce neuron loss and cognitive decline.^12^ Tau is a highly soluble and intrinsically disordered protein in its native form,^13,14^ even when bound to the microtubule surface.^15^ Yet, its self-assembly into amyloid-like fibrils and PHF requires folding of the protein leading to the characteristic cross β structure of amyloid fibers.^7^ Unlike Aβ which aggregates spontaneously *in vitro*,^16^ Tau requires a co-factor to self-assemble and form fibers.^17,18^ Negatively charged molecules have demonstrated the ability to trigger Tau aggregation, including RNA^19^ and sulphated glycosaminoglycans such as heparin sulfate,^20^ largely used for *in vitro* studies of Tau. However, a recent study has shown that the structure of heparin-induced fibers formed *in vitro* differs from fibers formed *in vivo*.^21^ In addition, several studies point to potential membrane-based mechanisms of tau aggregation.^22–25^

**Fig. 1:**
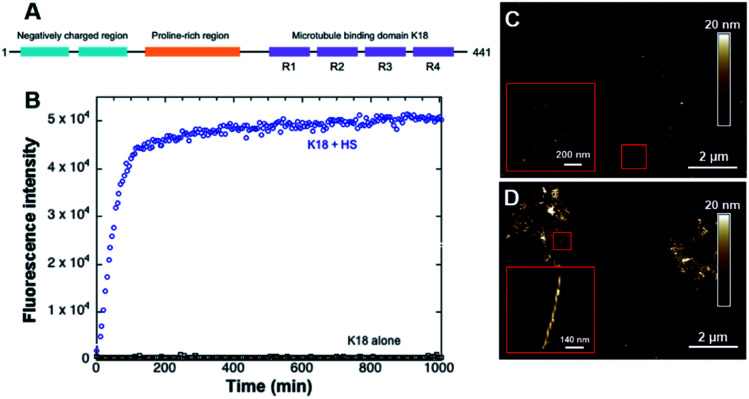
Structure of full-length Tau (A). Graph B represents ThT fluorescence assay curves obtained with 10 μM K18 without (black) and with (blue) 20 μM heparin sulfate (HS), with associated AFM images of K18 incubated alone (C) and of K18 fibers formed with HS as a cofactor (D).

The microtubule binding domain contains 3 to 4 repeat units (R1, R3, R4, and/or R2) and plays a decisive role in the aggregation process as it has been shown to induce the nucleation and constitutes the core of the PHF in AD.^26,27^ A conformational change from random coil to β-sheet has been locally observed in R2 and R3 repetition units, which indicates that the amyloid-like characteristics of the protein most likely stem from its microtubule-binding domain. In particular, two short portions of 6 amino acids within the R2 and R3 repeat units have been identified as initiators of the folding.^28,29^ Many studies have hence been focusing on two truncated tau constructs, K19 and K18, composed of three or four repeat domains that constitute the microtubule binding domain.^30,31^ The tendency of these peptides to aggregate is even more favorable than longer protein isoforms.^32^ K18 is a 12 kDa peptide which sequence constitutes the full microtubule binding domain (Fig. 1A).^33^ It shows a higher binding affinity to the microtubules^34^ and aggregation properties^14^ compared to K19.

Recently, the hypothesis of an interaction of Tau with membranes has been attracting increasing interest as the protein has been shown to interact with the membrane of neurons.^35–37^ Phospholipids have demonstrated the ability to initiate the aggregation^24,31,38,39^ and fibrillization^25^ of Tau or K18 *in vitro*. Furthermore, there is evidence of lipids in the fibers formed *in vivo*^40^ and *in vitro*.^25^ As Tau is an intracellular protein, current studies focus on lipids found in the membrane's inner leaflet, with which the protein most likely interacts.^38,39^ Electrostatic interactions are coined to be pivotal in triggering Tau fiber formation as only anionic molecules were shown to be efficient.^41^ Yet interactions with lipids have overall not been studied in detail, and there is still a lack of knowledge regarding how the nature of lipids modulates their interaction with the protein, and the underlying molecular modes of action.

In this work, Atomic Force Microscopy (AFM) was used to investigate the interactions of K18 with supported lipid bilayers (SLB) of different compositions. This approach is well suited for monitoring the peptide's interactions with membranes *in situ* with high lateral and vertical resolutions and in physiologically relevant conditions.^42,43^ We selected relevant lipids in order to assess the effect of charge, lipid packing and phase separation: POPC, the main lipid in mammal membranes, DOPC, DPPC and cholesterol, which are known to show lateral phase separation,^43^ and two anionic lipids of the inner neuronal membrane, POPS or PIP_2_,^44,45^ which has recently been identified as a co-factor in K18 aggregation.^25^ It is shown that K18 peptide can have detrimental effects on zwitterionic membranes as SLB solubilization. Interestingly, the peptide seems sensitive to the lipid packing density as only liquid disordered (ld) phases were significantly solubilized. K18 aggregation on SLB was only observed in presence of negatively charged lipids POPS and PIP2 included in POPC bilayers. However, fibers were only observed on POPC-PIP_2_ SLB, suggesting a specific role of PIP_2_ in the initiation of the fibrillization process. This work highlights that the behavior of K18 on lipidic membranes is modulated by lipid composition and lipid properties.

## Results

As a control, fluorescence assays were carried out with and without the cofactor heparin sulfate to verify the fibrillization of K18 prior to experiments in the presence of lipids. This anionic polysaccharide has been shown to induce the assembly of K18 after a subsequent binding to the R2 and R3 repeat units.^28^ In absence of heparin sulfate, the peptide did not form fibers, as confirmed by ThT fluorescence assays (Fig. 1B) and AFM imaging (Fig. 1C). On the contrary, incubation with heparin led to the formation of amyloid fibers (Fig. 1B and D). AFM imaging in liquid (Fig. 1D) demonstrated a twisted morphology reminiscent of PHF.^46^ However, it was recently shown by double electron–electron resonance spectroscopy that the conformation of *in vivo* formed fibers was different from that of fibers formed *in vitro* with heparin sulfate as a co-factor.^21^ This suggests other fibrillization pathways that require further investigation, notably a potential implication of membrane lipids in the fibril formation.

### Effect of K18 on single zwitterionic lipids SLB

First, we focused on model membranes composed of single zwitterionic lipids, POPC, as it constitutes the main lipid in mammal membranes, DOPC or DPPC. Control experiments showed no significant change in the SLB when imaged alone in HEPES buffer (Fig. S1†), even after a few hours of imaging.

**Fig. 2:**
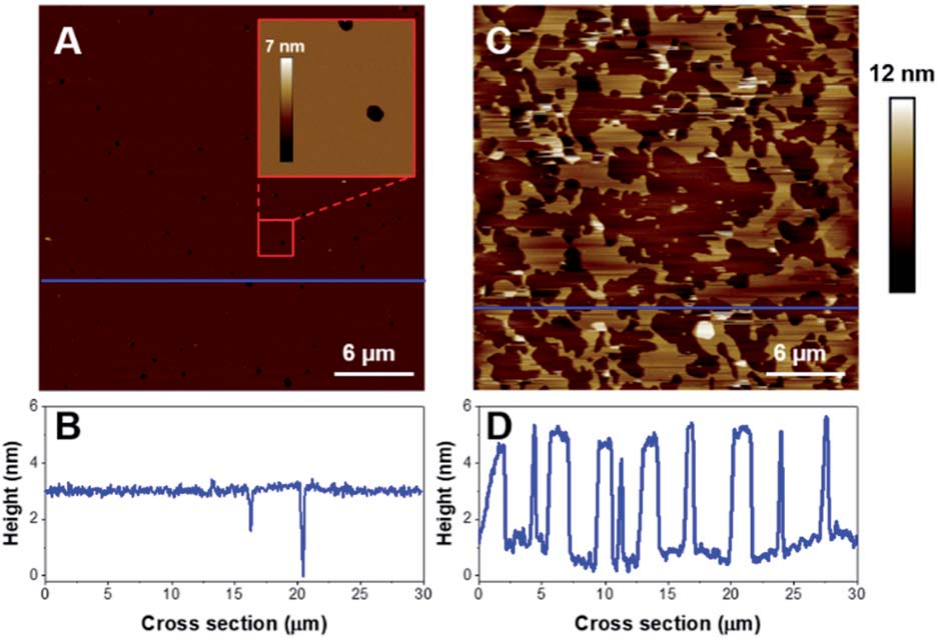
Effect of K18 on POPC SLB. 30 × 30 μm^2^ AFM images of pure POPC SLB and cross-section along the blue line before the addition of the peptide (A, B) and (C, D) after a 1 hour incubation with 1 μM of K18. Vertical scale is 0–12 nm.

**Fig. 3:**
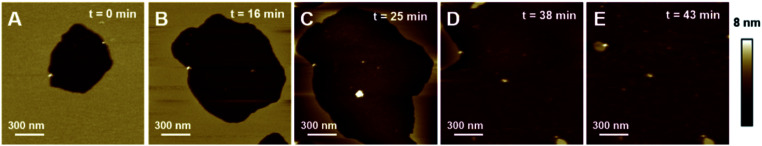
AFM images (1.5 × 1.5 μm^2^) of a POPC SLB incubated with 75 nM of K18. Image A represents a residual defect in the SLB before the peptide addition, and B to E were recorded after the addition of K18.

**Fig. 4:**
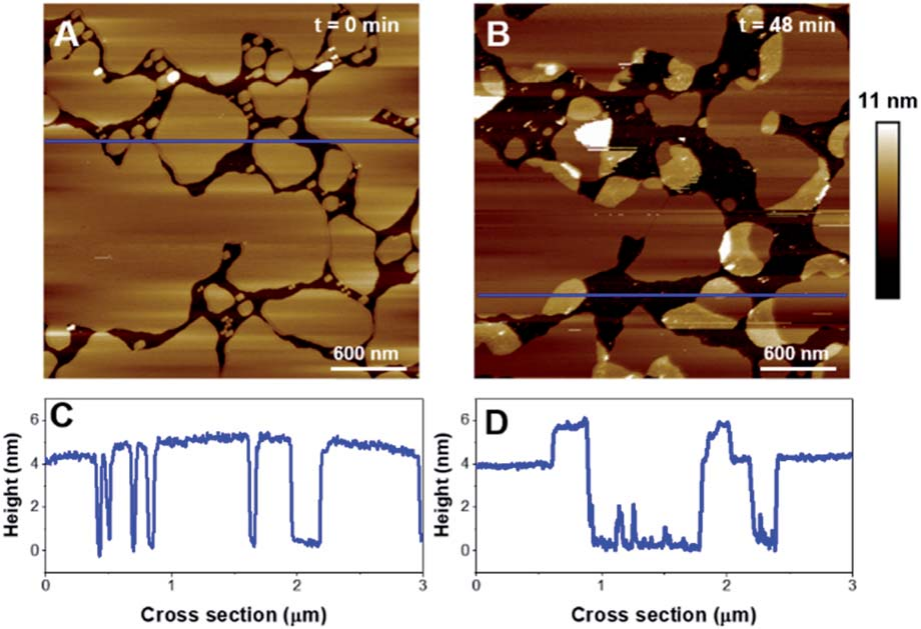
AFM images (3 × 3 μm^2^) of a DPPC supported lipid bilayer before (A) and after 48 minutes of incubation with 1 μM K18 (B). Graphs C and D represent the cross-sections corresponding to the blue dashed lines on A and B respectively.

The deposition of POPC SUVs led to the formation of a uniform SLB with a thickness of 3.6 ± 0.2 nm as shown in Fig. 2A. When K18 was added to the system at a concentration of 1 μM, the peptide provoked significant disrupting effects with the solubilization of a large proportion of the SLB after one hour of incubation (Fig. 2C and D). Similar behavior was observed for POPC bilayers incubated with a lower peptide concentration (75 nM, Fig. 3). When focusing on static features of the SLB such as a residual defect, the solubilization of the membrane was observed starting from the edges as depicted on the AFM images on Fig. 3A–E. No significant difference was observed regarding the timescale of the membrane solubilization despite the lower concentration of K18, which could be explained by potential local inhomogeneities in peptide concentrations depending on the proximity of the scanning site to the injection site. In order to explore the influence of different membranes properties, SLBs in the gel phase were prepared to determine how K18 may interact with a highly ordered membrane. The prepared monophasic DPPC SLB was 4.5 ± 0.1 nm thick, in good agreement with the longer extension of the acyl chains (Fig. 4A and C). After 48 minutes of incubation, K18 demonstrated a very limited ability to solubilize gel-phase SLB (Fig. 4B and D) compared to POPC fluid bilayers. However, a thickness increase at the edges of the bilayer domains is noticed, which could be due to the accumulation of the peptide at the bilayer edges, within, on or under the membranes.

### Effect of K18 on biphasic lipids SLB containing zwitterionic lipids

**Fig. 5:**
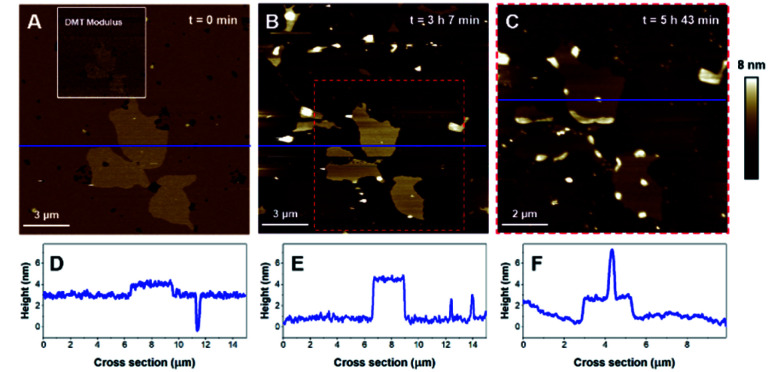
AFM images of a DOPC/DPPC bilayer (1 : 1). Images A and B (15 × 15 μm^2^) show the SLB before and after 3 hours of incubation with K18 (1 μM). Image C displays the area identified by a red dashed square on panel B, after ∼6 hours incubation. Graphs D, E and F are the cross-sections corresponding to the blue lines on A, B, and C respectively.

**Fig. 6:**
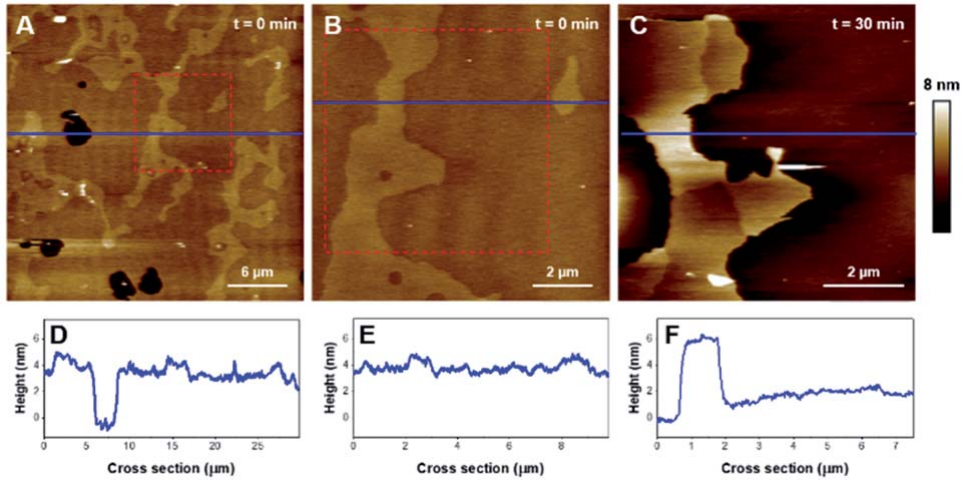
AFM images of a DOPC/DPPC/cholesterol bilayer (1 : 1 : 1). Image A (30 × 30 μm^2^) displays the SLB and image B represents the 10 × 10 μm^2^ area identified by the red square on A. Image C is the same area, recorded after a 30 min incubation with K18 (1 μm).

Then, the impact of the presence of gel/fluid lipid domains on the activity of K18 was characterized using biphasic SLBs made of an equimolar mixture of DOPC and DPPC, showing lateral phase separation at room temperature,^43^ as observed on Fig. 5A–D, as well as SLBs of DOPC–DPPC–cholesterol (1–1–1) (Fig. 6). Cholesterol is a major component of the plasma membrane, representing up to 50% of the lipids in neural membranes.^44^ In addition, it is known to greatly modulate the properties of membranes, in particular the lipid packing and order, as well as the membrane permeability.^47^ The higher domains attributed to DPPC were stiffer than the lower phase attributed to DOPC (Fig. 5A), in agreement with literature.^48,49^ Control experiments confirmed that the model membranes did not undergo any significant change when no peptide was added for incubation periods of up to 7 hours (Fig. S2†).

Regarding the DOPC–DPPC SLB, the thinner liquid disordered phase (DOPC-rich) exhibited a thickness of 3.6 ± 0.2 nm while the thicker gel phase (DPPC-rich) domains were 4.6 ± 0.3 nm thick, a thickness complying with the pure DPPC SLB that were previously studied. After a 3 hour incubation with K18 (Fig. 5B, E), a substantial solubilization of the ld-phase fraction of the SLB is observed while the gel phase domains seemed to remain mostly intact over this time window. After a 6 hour incubation (Fig. 5C, F), an increased thickness of the SLB edges by several nanometers was observed, similarly to the previous results obtained with the DPPC monophasic gel-phase bilayers. The thickening for the DOPC/DPPC system occurred hours after the one of pure DPPC bilayers. This difference may be attributed to a lower concentration of free K18 in solution as some of the peptides could be associated with lipid fragments resulting from the substantial extraction of the liquid disordered phase domains. When incubated with DOPC–DPPC SLBs, the K18 peptide did not show any form of aggregation on the exposed mica surface even after longer incubation periods (Fig. 5C). Yet, the injection of the K18 peptide alone on bare mica has shown that the peptide accumulates on the solid surface and small aggregates were observed after 2 h 45 of incubation (see Fig. S3†). This suggests that K18 preferentially interacts with lipids when a SLB is present on the mica surface.

Then, the effects of the peptide on a ternary mixture including cholesterol have been investigated. DOPC/DPPC/cholesterol (1 : 1 : 1) mixtures also led to phase separation and the formation of ld and lo domains. The SLB formed with this ternary mixture, displayed on Fig. 6A and B (plus corresponding cross-sections in Fig. 6D, E), was 4.0 ± 0.2 nm thick and displayed a height difference of 0.9 ± 0.1 nm between the ld and lo domains, features comparable to previous reports.^50^ After incubation with K18, the ld phase was rapidly solubilized and extracted from the surface, leaving only the lo phase on the mica substrate (Fig. 6C, F). The nature and the kinetics of the effects were comparable to those observed with the DOPC/DPPC binary system, suggesting that K18 detrimental effects on the membrane are not influenced by the presence of the sterol.

In order to provide clearer evidence that the Lo phase is not affected, or at least not as much as the Ld phase, by the membrane disruption process induced by K18, we have quantified the area covered by the Lo phase and its evolution with time for biphasic SLBs. For the DOPC-DPPC SLB figured in Fig. 5, the area of interest was initially covered at 98% by lipids, with higher domains (above 4.5 nm) occupying (Fig. 5A) ∼11.5% of the covered area. After 3 h of incubation, the area of interest is covered only at 14.3% by lipids, all of which presenting a thickness above 4.5 nm. The Lo domain, with its recognizable shape, initially represented 25 μm^2^, and shows a 30% decrease in size, with 17.8 μm^2^. In contrast, the Ld phase, which initially covered 86.5% of the area with a height <4 nm, has completely disappeared from the mica surface. Similarly, between 3 h 7 min and 5 h 43 min of incubation, the percentage of the mica surface covered in lipids went from 21% to 19%, showing again very little disappearance of the Lo domain. Similar effects are observed for DOPC–DPPC–Chol SLB. In Fig. 6B, the higher domains of the DOPC–DPPC–Chol SLB account for 23.5% of the red squared area of interest, completely covered by the SLB. This same area after 30 min of incubation with K18 shows a 67% surface coverage decrease, which accounts for the majority of the Ld phase. The Lo domain's shape remains recognizable, despite changes in the morphology.

### Effect of K18 on SLB containing anionic lipids

Finally, the impact of two anionic lipids, POPS and PIP_2_, on the K18/membrane interaction have been investigated. Phosphatidylserines are important components of the inner leaflet of the plasma membrane, representing about 10 to 20 mol% of its lipid composition. These lipids are also proposed to be involved in apoptosis.^44^ PIP_2_ is essentially located in the inner leaflet of the eukaryotic cell membrane, plays a fundamental role in regulatory functions^45^ and interacts specifically with intracellular proteins.^51^ Both POPS and PIP_2_ were embedded at 20% in a POPC matrix. This percentage is higher than the natural occurrence presence of these lipids in neuronal membranes,^44^ in order to amplify even weak interactions and to be able to detect them at the time scale of our experiments. Recently, the ability of PIP_2_ to promote K18 peptide fibrillization has been shown *in vitro* and the lipid was found to be a cofactor and a co-aggregating agent of K18.^25^ Control experiments confirmed that model membranes did not undergo any significant change when no peptide was added (Fig. S4†).

**Fig. 7:**
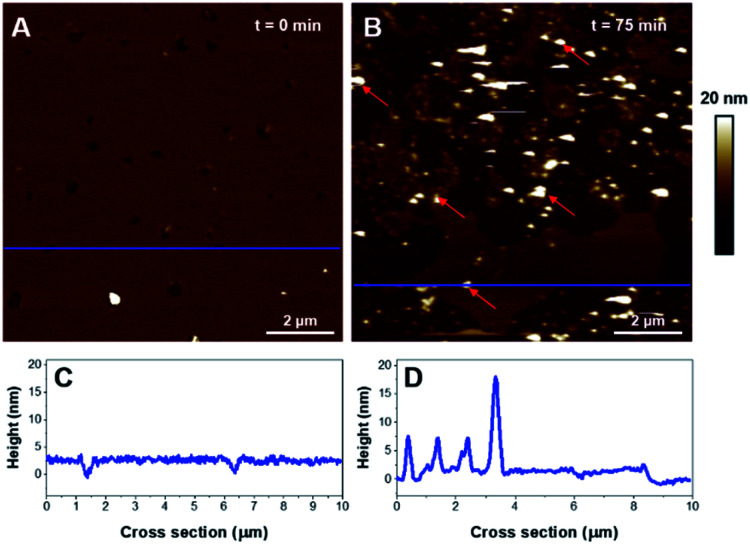
AFM images of a POPC/POPS bilayer (4 : 1). Images A and B show the SLB before incubation and after a 75 min incubation with 1 μM K18, respectively. Graphs C, D are the cross-sections corresponding to the blue dashed lines on A and B respectively.

Then the impact of K18 on POPC/POPS (4 : 1) bilayers was determined. The effective deposition of a negatively charged supported lipid bilayer generally requires the use of Ca^2+^ in order to screen the negative mica surface.^52^ However, calcium ions are known to bridge negatively charged lipid polar heads, restricting membrane diffusion.^53^ Moreover it was demonstrated that the presence of such divalent cations could induce the formation of laterally separated domains in SLB, a phenomenon that was shown to be dependent on the Ca^2+^ concentration.^54,55^ To limit the influence of Ca^2+^, the POPC/POPS (4 : 1) SUVs were prepared in an Hepes buffer void of Ca^2+^ and a small volume (20 μL) of Ca^2+^ solution (1 mM) was added independently to recover the mica surface, in prior to adding the SUV suspension. The disc was then washed several times with buffer. As observed on Fig. 7A, a monophasic SLB was obtained, with 3.2 ± 0.3 nm thickness (Fig. 7C). No evidence of domains was detected suggesting the mixture was homogeneous. The addition of K18 led to an important solubilization of the bilayer and concurrently to the formation of large aggregates of very heterogeneous thicknesses (see red arrows on Fig. 7B and D). These aggregates are hypothesized to be aggregates formed by the peptide or a mixture of K18 and lipids.

**Fig. 8:**
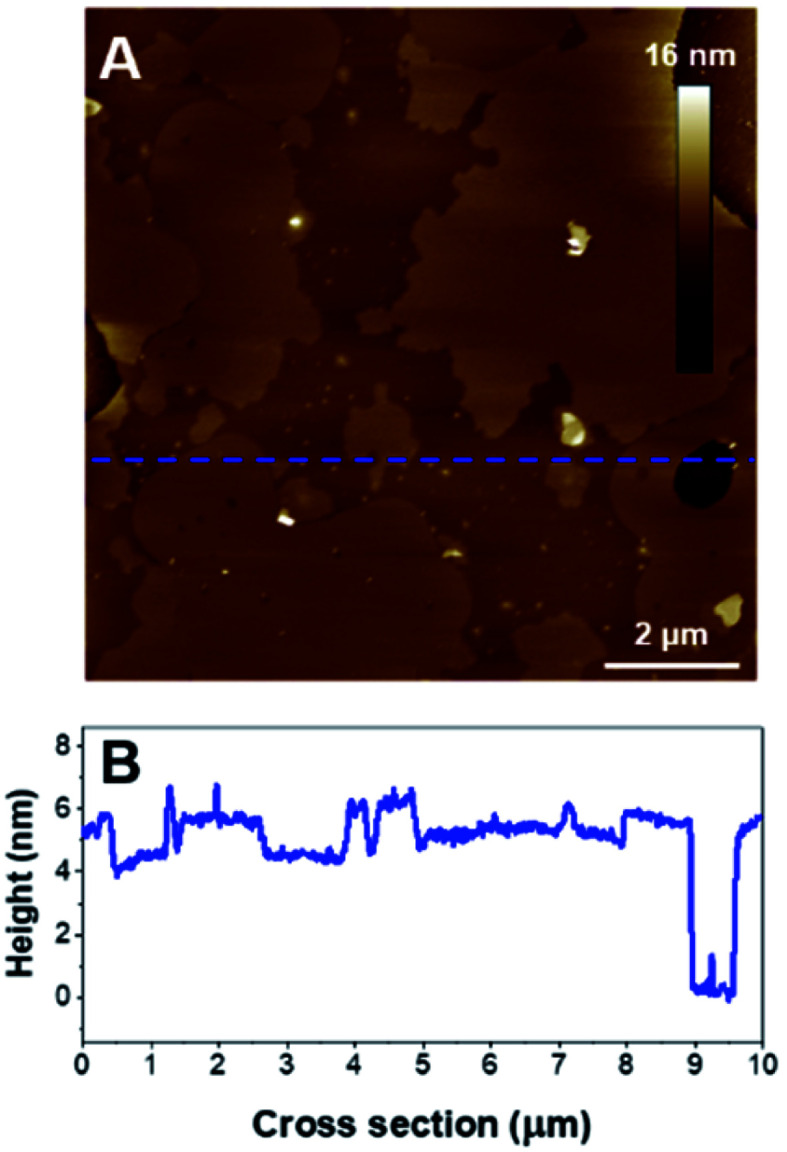
(A) AFM height image (10 × 10 μm^2^) of a POPC/PIP_2_ bilayer (4 : 1). B is the cross section associated to the blue dashed line on A.

As for the previous composition, the formation of SLB with PIP_2_-containing vesicles required a slightly different protocol compared to zwitterionic lipids. PIP_2_ bares 3 negative charges at physiological pH, which should lead to important electrostatic repulsions with the mica surface. It has been shown that acidic buffer conditions were efficient for the formation of SLBs of comparable compositions on negatively charged surfaces such as silicon.^56^ The SUV suspension was then prepared in a citrate buffer adjusted to pH 3 in order to protonate the phosphate groups of the PIP_2_ headgroup and favor the deposition of vesicles. The disc was rinsed with pH 7.4 buffer to carry out the incubation with K18 in more physiological conditions. Interestingly, as seen on Fig. 8A, B, the lipid mixture formed a continuous SLB that exhibited an important phase separation. The cross-section measurements indicated that the mean SLB thickness was 4.8 ± 0.3 nm and a level difference of 1.3 ± 0.2 nm between the phases. Since PIP_2_ has demonstrated the ability to form clusters *in vitro*^57,58^ and due to its more voluminous headgroup, the thicker domains were assumed to be enriched in phosphatidylinositol.

**Fig. 9:**
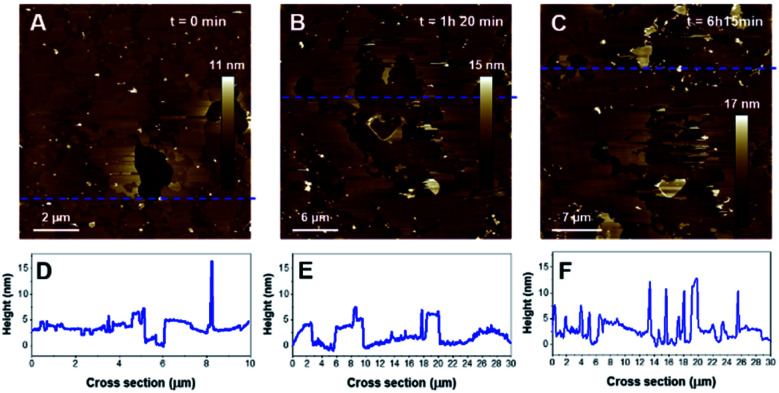
AFM images of a POPC/PIP_2_ (4 : 1). Images A, B and C show the SLB before, after 1 hour and 20 minutes and after 6 hours and 15 minutes incubation with K18 (1 μM). Graphs D, E and F are the cross-sections corresponding to the blue dashed lines on A, B, C respectively.

**Fig. 10:**
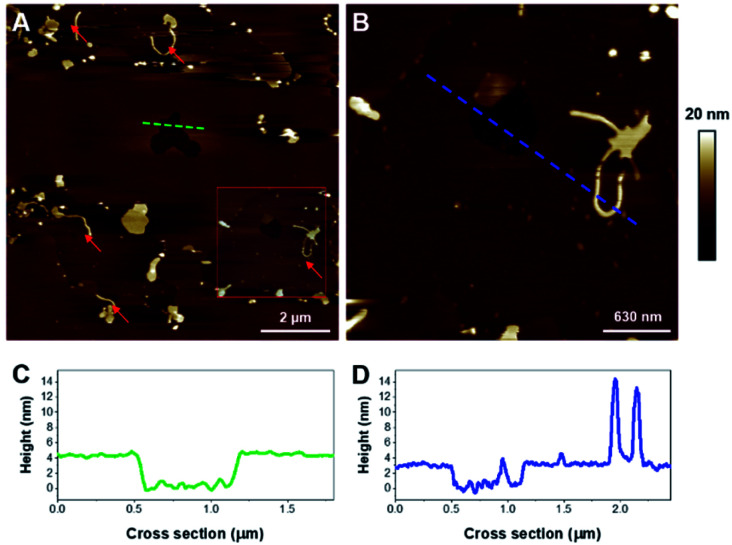
AFM images of the POPC/PIP_2_ after a 6 h incubation with K18 (1 μM) (A, B) with associated cross-sections (C, D). Formed filiform structures are pointed out with red arrows on A. Image B corresponds to the red frame figured on panel A.

When added, K18 induced an important disruption of the SLB with membrane solubilization, but also a significant reorganization of the domains as the morphology of the lipid domains dramatically changed after a 80 min incubation with K18 (Fig. 9B). Parts of the membrane were fragmented as it can be observed on the cross section (Fig. 9E). Interestingly, after a 6 hour incubation, the formation of aggregates was observed on the SLB, with thicknesses ∼10 nm (Fig. 9, upper right corner notably). When zooming in (Fig. 10), fibrils were observed as indicated by red arrows (Fig. 10A). The fibers seemed to have grown from aggregates on membranes (Fig. 10B and D). The thickness of the fibers was estimated to 10.3 ± 0.5 nm (*n* = 6). Interestingly, the highest point of the SLB after 6 h incubation was ∼4.1 ± 0.3 nm (Fig. 10C), lower than the initial thickness. In addition, fibrils seem to be assembled on lipid surfaces with thicknesses ∼2.7 ± 0.5 nm, which is consistent with the thickness of a lipid monolayer (Fig. 10B, D), and suggests lipid removal.

## Discussion

In this work, the behavior of K18, a fragment of Tau, was studied in presence of SLB with various lipid compositions to understand how the nature of membrane lipids may drive the interactions with the peptide, leading or not to peptide aggregation. It is shown that the interaction of K18 with membranes is dependent of the SLB lipid composition.

### K18 solubilizes zwitterionic fluid phases in SLB

On monophasic and biphasic membranes made of zwitterionic lipids, the interaction of K18 with the SLB has been shown to be detrimental, leading to an important membrane solubilization of fluid phases. This effect was typically observed on all compositions containing a liquid disordered phase formed by POPC or DOPC. The solubilization of the fluid phase in phospholipid membranes appeared to be initiated at the edges of the SLB with a progressive lateral solubilization of the SLB from residual defects as already observed in other studies.^59–61^ The presence of cholesterol, known to increase the packing density of bilayers by condensing the hydrocarbon chains within the fluid phase,^47^ did not prevent the membrane disruption by the peptide.

This disruption might result from a lateral insertion of K18 and could explain the increased thickness observed at the edges of DPPC domains. The peptide somehow could not induce enough disruption to solubilize the membranes, probably due to a higher packing density of lipids in both gel and lo phases. Edges in SLB are often considered to constitute favorable anchoring sites for proteins or peptides. The insertion of alkaline phosphatase was for instance observed at the edges of gel-phase patches in DOPC-SM bilayers,^62^ leading to increased thickness. These phenomena involving SLB edge targeting are reminiscent of the actions of antimicrobial amphipathic peptides that display comparable propensities to solubilize membranes.^59,60^ In a comprehensive study, Henderson *et al.* observed the common property of such antimicrobial peptides to solubilize membranes from the edges of zwitterionic SLBs, a phenomenon leading to the lowering of line tension.^63^ No evidence of peptide accumulation was observed on or near zwitterionic lipid membranes. This observation suggests the potential formation of mixed K18/lipid complexes that would be released in solution, a hypothesis previously proposed for other amyloid peptides.^50,61^ The analysis of the supernatant after incubation did not reveal any evidence of fibers. This observation correlates with the absence of ThT fluorescence that have been reported with zwitterionic vesicles incubated with K18.^25^ Compared to the different mechanisms of membrane disruption that have been proposed, the disrupting effects appears to be associated with a detergent-like model.^64^

### Negatively charged lipids promote K18 aggregation onto the SLB

K18 contains 5 histidine and 19 lysine residues within its sequence.^28^ At physiological pH, histidine is neutral but lysine bears a positive charge, pivotal for the binding activity of Tau protein to the microtubules.^34,65^ The net charge of K18 was measured to be +9 at pH 7.^14^ To explore the potential electrostatic affinity of K18 with lipids, two binary systems with controlled compositions were used, focusing on two negatively charged lipids from the inner leaflet of the neuronal membrane: POPS and PIP_2_.

In case of POPS-containing SLBs, the injection of K18 induced a solubilization of the membrane but also led to the formation of visible aggregates of varied heights (5–30 nm) on the surface, after only 75 min of incubation. In contrast to previously investigated systems, this was the first evidence of K18 aggregation and deposition on a SLB. Recently, it was proposed that such aggregation of K18 was occurring when a critical concentration of peptide was reached, based on results obtained from anionic vesicles containing POPS^31^ or from fatty acid micelles.^39^ The ability of anionic agents to promote Tau protein aggregation is thought to stem from the neutralization of the positive charges contained within the microtubule associated domain. The negatively charged surface of the SLB containing the phosphatidylserine was shown to be a factor of aggregation for K18, which corroborates the importance of electrostatic interactions for promoting a locally increased concentration of K18, thus favoring its aggregation. A recent AFM study corroborates the importance of electrostatic interactions as the association of Tau protein on SLB prepared from total brain extract was shown to be a sodium cation-dependent process.^24^ The binding of K18 onto such mixed SLB was proposed to be assigned to POPS.^38^ Interestingly this study also reported the disruption of POPS-containing vesicles leading to the formation of K18/lipid complexes. No fibrillization was reported in any of these, similarly to our observations. Tau-induced pore formation was previously observed in DOPS lipid bilayers,^66^ but we did not observe this structuration in our lipid bilayers containing anionic lipids.

In contrast with POPS, our observations reveal that PIP_2_-containing SLB not only initiated the aggregation of K18 but also promoted the formation of fibers, in what seems to be a membrane-supported process. This would be in good agreement with recent *in situ* synchrotron grazing-incidence X-ray diffraction results, which showed that both hTau40 and K18 in interaction with anionic membranes of DMPG assembled into misfolded oligomers with a β-sheet conformation at the membrane surface, associated with membrane disruption.^67^ It was however difficult to identify if the fibrils formed on a particular phase of the binary mixture system as the incubation with the peptide greatly affected the morphology of the domains. In any case, the fibers seemed to have grown from membrane-supported aggregates, which then appeared to act as nucleation sites. The peculiar ability of PIP_2_ to form clusters with positively charged peptides have been reported in membranes.^58^ This could support the hypothesis of PIP_2_ seeding sites that might have promoted fibril growth. A previously study using tip-enhanced Raman spectroscopy (TERS) demonstrated that PIP_2_ was incorporated into K18 fibers.^25^ The fibers formed in our system over less than 6 hours of incubation and exhibited a thickness of about 10 nm. This value slightly differs from the diameters of amyloid fibrils obtained with PIP_2_-induced fibrillization in solution, which were in the 3–7 nm range. Heparin-induced tau filaments have been reported to have 15- or 20 nm diameter, for straight or twisted filaments respectively.^20^ Our observation that fibrils seem to be assembled on lipid surfaces with thicknesses consistent with lipid monolayers suggests lipid removal and would concord with previous data showing lipid recruitment in the fibers.^25^

In our study, K18 fibrillization was only observed for PIP_2_-containing SLBs, which highlights the importance of lipid composition in the fibrillization process. This finding also demonstrates the necessity to use more biologically relevant systems for biophysical studies of Tau fibrillization as the observed fibers morphologically differ from heparin-induced filaments usually used in biophysical studies. This is in good agreement with recent studies, which have shown that the structure of heparin-induced filaments differ from filaments extracted from a diseased patient^21,68^ and that PIP_2_-induced fibers are rich in β-sheet contrarily to heparin-induced aggregation products.^25^ The next step to complement this study related to the aggregation of K18 will be to elucidate the toxicity mechanisms of K18 and Tau at the cellular^69,70^ or tissue level as some questions are still remaining on how they contribute to cell death and dysfunction.

## Experimental

### Materials

1-Palmitoyl-2-oleoyl-*sn*-glycero-3-phosphocholine (POPC), 1-palmitoyl-2-oleoyl-*sn*-glycero-3-phospho-l-serine (POPS), 1,2-oleoyl-*sn*-glycero-3-phosphocholine (DOPC), 1,2-dipalmitoyl-*sn*-glycero-3-phosphocholine (DPPC), 1,2-dioleoyl-*sn*-glycero-3-phospho-(1′-myo-inositol-4′,5′-bisphosphate (PIP_2_, isolated from porcine brain) and cholesterol were purchased from Avanti Polar Lipids (Alabaster, AL, USA). The phospholipids were received as organic solutions (chloroform and chloroform/methanol/water for PIP_2_ (20 : 9 : 1 vol/vol)) while cholesterol was obtained as a powder. 4-(2-Hydroxyethyl)-1-piperazineethanesulfonic acid (Hepes), sodium citrate tribasic dihydrate, calcium chloride (CaCl_2_) sodium chloride (NaCl) and heparin sulfate were obtained from Sigma (Steinheim, Germany). Aqueous solutions were all prepared with ultra-pure water (18 MΩ cm).

### Peptide production

pNG2 K18 (kindly provided by Pr. E. Mandelkow) was used to transform *Escherichia coli* C41(DE3) (F- ompT hsdSB (rB- mB-) gal dcm (DE3)). Several transformants were grown on 120 mL LB + 1% dextrose, 100 mg L^−1^ ampicillin. When the culture reached an optical density OD_650_ = 0.52, 10 mL was added to 990 mL of ZYM 5052 medium (1% *N-Z*-amine, 0.5% yeast extract, 25 mM Na_2_HPO_4_, 25 mM KH_2_PO_4_, 50 mM NH_4_Cl, 5 mM Na_2_SO_4_, 2 mM MgSO_4_, 0.5% glycerol, 0.05% dextrose, 0.2% lactose) containing 100 mg L^−1^ ampicillin and incubated overnight at 37 °C. After centrifugation, cell pellets were suspended in 50 mL of 2-(*N*-morpholino)ethanesulfonic acid (MES) pH 6.8 (20 mM), NaCl (500 mM), ethylenediaminetetraacetic acid (EDTA, 1 mM) phenylmethylsulfonyl fluoride (PMSF, 1 mM), benzamidine (2 mM), and dithiothreitol (DTT, 5 mM), sonicated four times (1 min cycles on ice; output 5, 50% duty cycle) and then heated at 80 °C for 20 min. After centrifugation (30 min at 15 000 g), the supernatant was then dialyzed for at least 16 h at 4 °C against cation exchange buffer A (20 mM MES, EDTA 1 mM, NaCl 50 mM, DTT 1 mM, pH 6.8) with a Spectra/Por Dialysis Membrane (MWCO 3.5 kDa). The dialysate was then cleared (30 min, 15 000*g*), filtered through a 0.22 μm membrane and applied onto a HiTrap SP (GE Healthcare, France) equilibrated with the cation exchange buffer A. After washing with 25 mL of the same buffer, the protein was eluted with 25 mL of Buffer B (MES 20 mM, EDTA 1 mM, NaCl 150 mM, pH 6.8) and the 5 mL fractions containing most of K18 were pooled and concentrated by ultrafiltration devices (*e.g.*, Ultrafree, Millipore 5 kDa MWCO) to a final volume of 0.5 to 1 mL. Finally, the protein concentrate was applied onto a gel filtration column (Superdex-75) equilibrated in 100 mM Ammonium acetate containing 2-mercaptoethanol (0.1%). The fractions containing the pure protein were pooled, aliquoted, and lyophilized.

### Preparation of the lipid suspensions

Appropriate volumes of individual lipid solutions were mixed to obtain the desired molar ratio. Organic solvents were evaporated under a gentle stream of nitrogen gas to form lipid films. In case of the mixtures with PIP_2_, evaporation was carried out at 50 °C to ensure the miscibility of the lipids. To remove residual solvent traces, the lipid films were further dried in vacuum at room temperature overnight. They were then dissolved into a Hepes buffer (20 mM, 140 mM NaCl, pH 7.4). For PIP_2_ mixtures with POPC, a citrate buffer (20 mM, 140 mM NaCl, pH 3) was employed. The final concentration of the suspensions was ∼1 mg mL^−1^ and each suspension was thoroughly agitated at 50 °C for 30 minutes. The resulting multilamellar vesicle suspensions (MLV) were then submitted to 3 freeze and thaw cycles and sonicated in a bath (Fisher Scientific, Illkirch, France) at 50 °C until the suspensions reached clarity. The resulting suspensions of Small Unilamellar Vesicles (SUV) were then filtered using a polyethersulfone filter with 200 nm pores and stored in Eppendorf tubes at 4 °C for no longer than 2 weeks.

### Preparation of suspended lipid bilayers and addition of K18

A volume of 80 μL of a SUV suspension was deposited onto freshly cleaved 10 mm muscovite mica disks (Agar Scientific, Stansted, UK) previously heated to 75 °C and left to slowly cool down for 30 minutes to allow for vesicle deposition. The remaining volume was removed and the disk was rinsed several times with buffer (20 mM Hepes, 140 mM NaCl, pH 7.4). In case of the POPC/POPS mixture (4 : 1), a volume of 20 μL of a CaCl_2_ solution (1 mM) was first deposited onto the mica disk before adding the SUVs suspension. For the POPC/PIP_2_ mixture (4 : 1), rinsing with the Hepes buffer was preceded by a 30 minute incubation in a mildly acidic citrate buffer (20 mM, 140 mM NaCl, pH 4.5). For imaging convenience, the experimental volume was set to 100 μL. Once the SLB was characterized by AFM, an aliquot of K18 solution was added after having removed an equivalent volume of buffer to set the peptide total concentration to 1 μM.

### Atomic force microscopy

Peak Force Tapping (PFT) mode was used to perform AFM imaging of the different SLB on a Dimension FastScan system (Bruker, Santa Barbara, CA) in liquid conditions at room temperature. Silicon nitride cantilevers (ScanAsyst Fluid+, Bruker) with a resonance frequency of 150 kHz, a nominal spring constant of 0.7 N m^−1^, and a tip radius of 10 nm were used for this work and were calibrated for each experiment. Images were acquired with a scan rate of ∼1.0–1.5 Hz, with a force kept as low as possible (typically 0.5 nN or lower). Resolution was set to 512 pixels × 512 pixels and imaging gains were automatedly optimized by the software. Images were analyzed and processed with the Nanoscope Analysis 1.8 software (Bruker, Santa Barbara, USA). For the time-dependent studies, the same selected areas were investigated even if some small drifts could occur. SLB thickness were determined with the cross-section tool by measuring the difference in height between the mica and the top of the membranes, typically at residual defects in the SLB. Special care was dedicated to avoid potential dehydration of the bilayers. When not scanning, the samples were covered with a Petri dish to minimize evaporation. When necessary, Hepes buffer was added to maintain a constant incubation volume and avoid potential variations of peptide concentration. Experiments were repeated to ensure reproducibility of the observed effects. To be sure that the membrane disruption was not due to a time effect, a reference sample without adding K18 was analyzed over time for each condition. Whatever the membrane composition, the membrane samples were stable over long periods of time.

### Fluorescence assays

Fluorescence assays were carried out in standard 96-well flat bottom and low binding black plates (Greiner, Frickenhausen, Germany) on a Clariostar microplate reader (BMG Labtech, Offenburg, Germany), at 37 °C using wavelengths of 440 nm and 480 nm for Thioflavine T (ThT) excitation and emission respectively. Final concentrations were as follows: 20 μM ThT, 10 μM K18, and 20 μM heparin sulphate when present.

## Conclusions

In conclusion, using AFM, we have demonstrated that K18 detains a detergent-like membrane solubilizing ability and that the lipid composition modulates the peptide's behavior. Detrimental phenomena were observed to be driven by the edges of the SLB and to disrupt specifically liquid disordered phases. Our results show that aggregation or fibrillization is not a prerequisite for K18 to exert a deleterious action on model membranes such as SLB. This study also demonstrates the ability of membranes containing anionic lipids to promote the microtubule-binding domain aggregation and highlights the decisive role of PIP_2_ to initiate the formation of fibers.

## Author contributions

M. A., C. F., M. L., M. M. and S. L. participated to the scientific discussions, analysed the data and wrote the manuscript. M. A. and C. F. carried out the AFM experiments and fluorescence assays. S. L. and M. M. designed and coordinated the project. All authors read and approved the final manuscript.

## Conflicts of interest

There are no conflicts to declare.

## Supplementary Material

NA-003-D1NA00055A-s001
