# Supplementary material for: Interaction of Tau construct K18 with model lipid membranes
Source: Nanoscale Adv. 2021 Jun 17;3(14):4244–53. doi: 10.1039/d1na00055a (PMC9417262; doi:10.1039/d1na00055a)
Supplement: NA-003-D1NA00055A-s001 [file NA-003-D1NA00055A-s001.pdf]

Electronic Supplementary Material (ESI) for Nanoscale Advances.
This journal is © The Royal Society of Chemistry 2021

Supplementary material

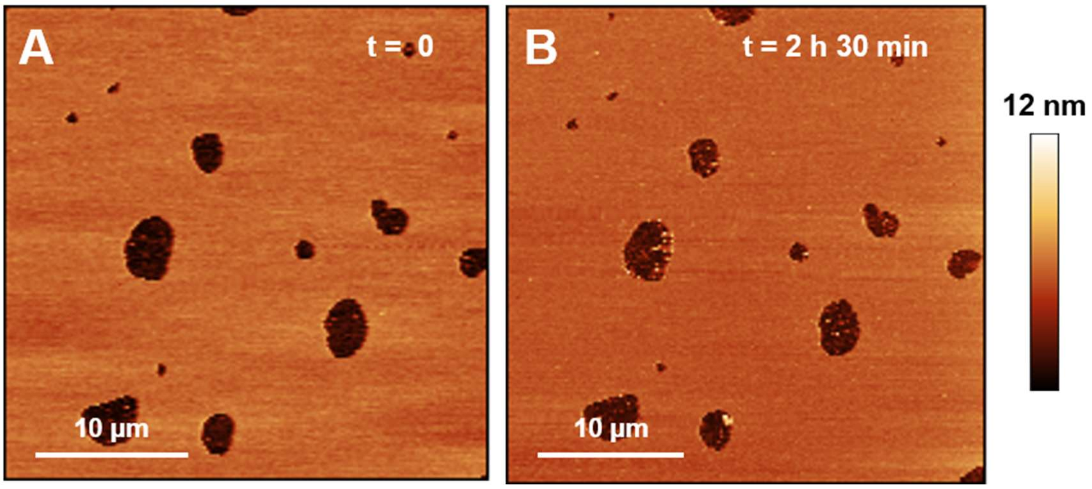

Figure S1 - AFM images of a pure POPC bilayer imaged at t = 0 (**A**) and after ~ 2h30 of imaging (**B**).

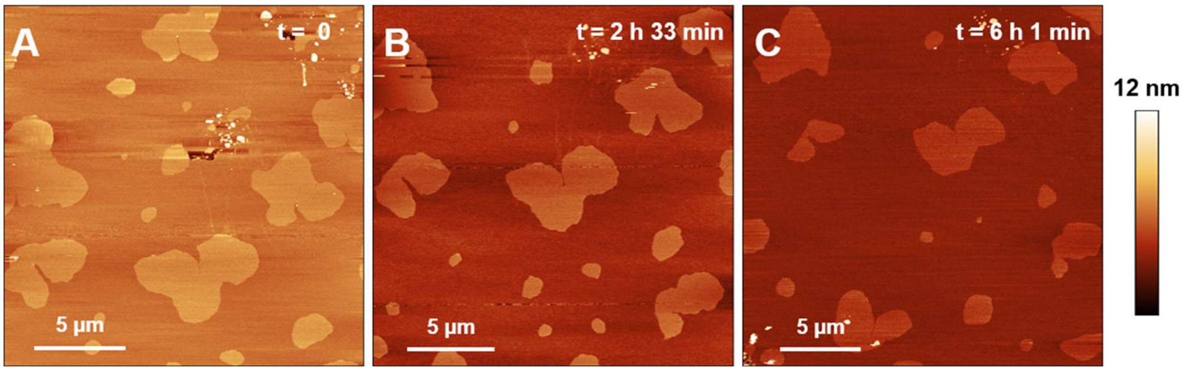

Figure S2 - AFM images of a DOPC/DPPC (1/1) bilayer at t = 0 (**A**) and after ~ 2h30 (**B**) and 6h of imaging (**C**).

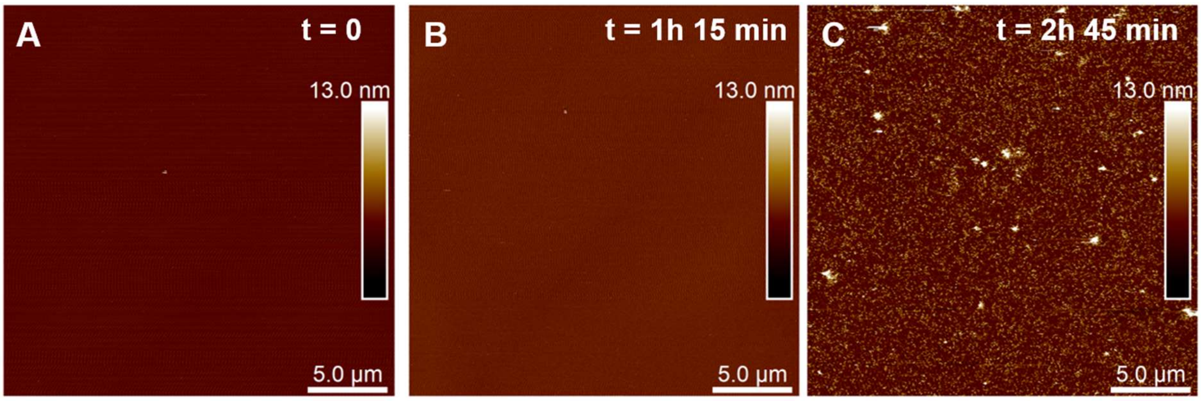

Figure S3 - AFM images of a mica substrate imaged at the time of injection of K18 (1 uM final concentration) (**A**) and after 1h15min (**B**) and 2h45min of incubation (**C**).

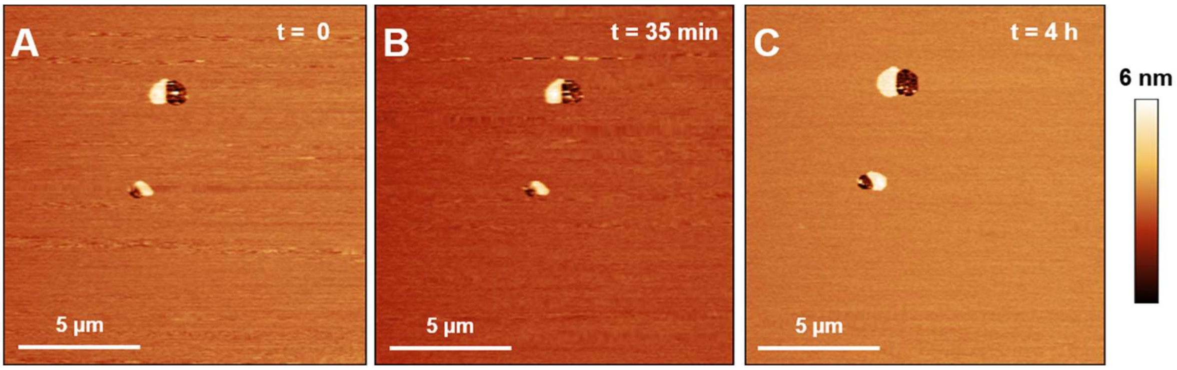

Figure S4 - AFM images of a POPC-POPS (4-1) bilayer at t = 0 (**A**) and after ~ 35 min (**B**) and 4 h of imaging (**C**).
